# Oak Leaves as a Raw Material for the Production of Alcoholic Fermented Beverages

**DOI:** 10.3390/foods13111641

**Published:** 2024-05-24

**Authors:** Tomas Pencak, Dani Dordevic, Sanja Ćavar Zeljković, Bohuslava Tremlova

**Affiliations:** 1 Department of Plant Origin Food Sciences, Faculty of Veterinary Hygiene and Ecology, University of Veterinary Sciences Brno, Palackeho tr. 1946/1, 612 42 Brno, Czech Republic; h23448@vfu.cz (T.P.);; 2 Centre of the Region Haná for Biotechnological and Agricultural Research, Department of Genetic Resources for Vegetables, Medicinal and Special Plants, Crop Research Institute, Šlechtitelů 29, 783 71 Olomouc, Czech Republic; sanja.cavar@upol.cz; 3 Czech Advanced Technology and Research Institute, Palacky University, Šlechtitelů 27, 783 71 Olomouc, Czech Republic

**Keywords:** *Quercus*, oak leaves, antioxidant capacity, polyphenols, alcoholic beverages, fermentation

## Abstract

This study aimed to point out the possible use of oak leaves (*Q. petraea*) in the production of fermented alcoholic beverages. Parameters such as antioxidant capacity, total phenolic content, phenolics and sugars were determined using spectrophotometric and chromatographic methods. pH values were also determined, and in the final product with a fermentation length of 85 days, the alcohol content was determined and sensory analysis performed. The antioxidant capacity of the beverage was lower compared to the infusions before fermentation, and its highest values were recorded in the leaf samples, in which the highest values of phenolic compounds and the total phenolic content were also recorded. A decrease in the content of total phenolics was recorded with the increasing length of fermentation in beverage samples. However, the fermentation process had a positive effect on the contents of some phenolic substances such as catechin, gallic acid and gallocatechin. Sensory analysis showed a higher acceptability of the fermented beverage without the addition of orange, which could be caused by the higher sugar content in these samples. Oak leaves therefore represent a suitable raw material for the production of a fermented alcoholic beverage, without the need to enrich the taste with other ingredients.

## 1. Introduction

Due to the increase in the human population, the current global food system will have to adapt, and this adaptation will probably include the increased consumption of edible wild foods, due to their richness in micronutrients and bioactive compounds [1]. A potential source of these bioactive compounds is the green leaves of plants, which can be used as a potential source for functional foods [2]. Oak leaves were used by native Mexicans, who used them to prepare foods and beverages such as corn tortillas with the addition of oak leaves, or they added oak leaves to eggs when making scrambled eggs. They also made an ancient fermented corn beverage called tesguino, with the addition of oak leaves to bring more flavor to the beverage [3]. Oak leaf infusions have been studied for their antioxidant, antimicrobial and gastroprotective effects [4], as well as their anti-inflammatory and anticarcinogenic activities [5]. However, despite their biological potential, products made from them present low acceptability for consumers, which is associated with the high bitterness of herbal infusions, reflected in the final quality of the product [6].

Other studies on the fermentation of oak leaf infusions have shown that antioxidant capacity, as well as sensory acceptability, were improved and the characteristic bitterness of these infusions was decreased after fermentation [7]. Other studies claim that the development of functional fermented beverages from plant-based unused resources, such as oak leaves, can represent an innovative area with high potential, and these functional beverages can provide protective effects on health due to the presence of phytochemicals and bioactive metabolites [8]. The production of herbal wines currently represents a benefit for the alcoholic beverage industry due to the presence of numerous secondary metabolites with many pharmaceutical characteristics. A large number of plants can be used to make herbal wine, wherein different parts, such as leaves, roots or flowers, can be used [9]. However, different wines made from different raw materials have a diverse composition of substances with an antioxidant effect, and the compositions and contents of phenolic substances depend on the raw materials and the production process [10].

Herbal wines made from unused plant agricultural resources, as well as from waste plant material, represent an alternative for use in the production of wines and alcoholic beverages, which, in addition to providing benefits for human health, also lead to a reduction in seasonal losses in plant production as well as a reduction in waste in the environment [11]. Wines enriched with herbal extracts represent a new trend in the production of alcoholic beverages [12], but they are still mainly fortified wines. However, the production of an alcoholic beverage using simple fermentation via leaf infusion can represent an affordable alternative using available unused plant raw materials. Therefore, the aim of this study was to explore oak leaves as a potential source for obtaining fermented alcoholic beverages, as an alternative to white wine.

## 2. Materials and Methods

### 2.1. Materials

*Q. petraea* leaves used for the production of herbal wine were obtained from trees located in the Brno-Jundrov area of Czech Republic 49°12′48.0″ N 16°32′47.9″ E in May 2022. Oranges and sugar were obtained from the market BILLA, spol. s r. o. Yeast and yeast nutrition were obtained from www.vinarskepotreby.cz (accessed on 1 May 2022). Infusion from oak leaves before and after fermentation as well as untreated fresh leaves and leached leaves were used in this study.

### 2.2. Preparation of Herb Infusions and Fermantation

**Figure 1:**
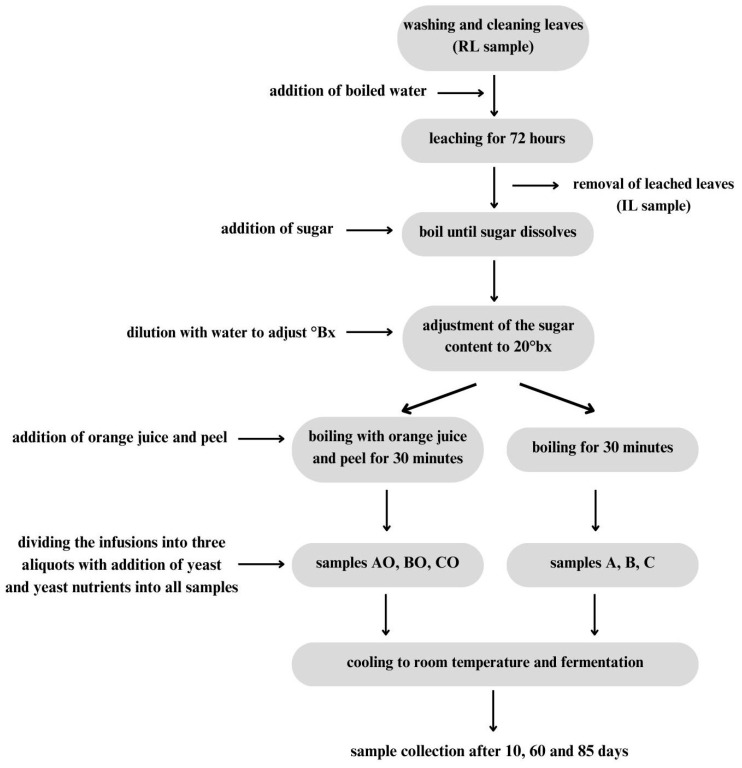
Alcoholic beverage production process.

The production process of fermented beverage is shown in Figure 1. Oak leaf infusions were prepared from 450 g of clean and washed leaves, which were poured with 6 L of boiled water. This infusion was allowed to stand for 72 h and then strained. Then, 2 kg of sugar was added to the obtained infusion and was left to boil until the sugar dissolved. The infusion was divided into two samples with a volume of 3 L each; 100 mL of orange juice and 50 g of orange peel were then added to one of these samples. Then, 2 L of water was added to each infusion to adjust the sugar content from the original 33 °Bx to 20 °Bx, and the infusions were then left to boil for 30 min. Both infusions were allowed to cool at room temperature, divided into three different samples and poured into bottles with a volume of 730 mL; 0.146 g of three types of yeast and 0.146 g of nutrient for yeast were added to all bottles. After adding the yeast and nutrients, the bottles were closed and left to ferment at room temperature. Samples were taken after 10, 60 and 85 days of fermentation, and then frozen. The samples’ descriptions are given in Table 1 and Table 2.

### 2.3. Preparation of Extracts for Spectrophotometric Determinations

For the preparation of the extracts used as part of the ABTS, DPPH, CUPRAC and total phenolic content methods, the following methodology was applied. For the preparation of the extracts of solid leaf samples, 0.1 g of the sample was weighed into dark test tubes, to which 20 mL of ethanol and water (1:1) was added, and this was extracted for 30 min in an ultrasound water bath and then filtrated. For the preparation of the extracts of liquid samples, 0.1 mL of sample was added instead of 0.1 g. This was followed by the same procedure as used in the case of a solid sample [13].

### 2.4. ABTS 2,2’-azinobis(3-ethyl-2,3-dihydrobenzothiazol-6-sulfonate)

At 12 to 16 h before the measurement, a reaction mixture was created by mixing 10 mL of ABTS solution with 10 mL of potassium peroxodisulfate solution, and the mixture was left to react in the dark. Before use, the reaction solution was diluted so that the resulting absorbance was 0.7. Before the measurement, 1980 μL of ABTS reaction solution was mixed with 20 μL of extract in 10 mL test tubes and left to react for 5 min in the dark. The absorbance was measured at a wavelength of 735 nm [13].

The results were calculated according to the following formula:

ABTS (%) = [(Abs_ABTS_ − Abs_sample_)/Abs_ABTS_] × 100

### 2.5. DPPH (2,2-diphenyl-1-picryl-hydrazyl)

Then, 3 mL was taken from the filtered extract, to which 1 mL of 0.1 mM DPPH ethanol solution was added. Blank solution was prepared in the same way using 3 mL of ethanol and 1 mL of 0.1 mM DPPH ethanol solution. Prepared solutions were subsequently shaken and incubated in the dark for 30 min. After 30 min of incubation, the absorbance was measured at a wavelength of 517 nm [13].

The results were calculated according to the following formula:

DPPH (%) = [(Abs_DPPH_ − Abs_sample_)/Abs_DPPH_] × 100

### 2.6. CUPRAC (CUPric Reducing Antioxidant Capacity)

Here, 1 mL of extract was mixed in a test tube together with 1 mL of Cooper solution, 1 mL of Neocuproin and 1 mL of buffer with pH 7. The sample was left to incubate in the dark for 1 h and measured at a wavelength of 450 nm against a blind sample. To prepare a blank sample, 2 mL of Cooper’s solution, 2 mL of Neocuproin, 2 mL of buffer and 2.2 mL of solvent were pipetted into the test tube. The results are expressed as µmol of Trolox per gram of sample [13].

### 2.7. Total Phenolic Content

Here, 1 mL of the filtered extract was mixed with 5 mL of 10% Folin–Ciocalteu solution and 4 mL of 7.5% Na_2_CO_3_. The sample was incubated for 30 min in a 25 mL volumetric flask in a dark environment. After incubation, the flask was topped up with distilled water up to the mark and the prepared sample was measured at a wavelength of 765 nm against a blank sample (1 mL of water was added instead of the sample). The results were expressed as gallic acid equivalents (GAE) per mg of sample [14].

### 2.8. Determination of Phenolics by UHPLC-MS/MS

For quantifying the contents of the phenolic compounds in raw and leached leaf samples, homogenized plant material (10 mg) was mixed with 1 mL of 80% MeOH, sonicated for 10 min in an ultrasonic bath and centrifuged at 14,500× *g*; the supernatant was transferred into the new vial and kept at −20 °C until the analysis was performed [15]. Liquid samples of infusions and leaf beverages were injected into a reversed phase column (Acquity BEH C_18_, 1.7 μm, 3.0 m × 150 mm, Waters, Milford, MA, USA) with an appropriate pre-column. The column temperature was maintained at 40 °C. The mobile phase contained 10 mmol/L formic acid in water (A) and acetonitrile (B) at a flow rate of 0.4 mL/min. The linear gradient consisted of 5% B for 3 min, 5–25% B for the next 4 min, 25–30% B for 6 min, 30–35% B for the next 4 min, 35–60% B for 6 min, 60–100% for the next 4 min, then isocratic for 1.5 min, back to 5% B within 0.1 min, and equilibration for 3.4 min. During the chromatographic run, the back pressure ranged from 45 to 50 MPa. Argon was used as a collision gas and nitrogen as a nebulizing gas. The analysis was performed in multiple reaction monitoring (MRM) mode [16].

### 2.9. Sugar Determination HPLC-ELSD Method

In the analysis of the three sugars glucose, fructose and sucrose, liquid samples were used for determination and applied directly on the HPLC. After the injection of the samples, the sugars were separated on a Rezex RCM monosaccharide Ca^+^ column (300 mm × 7.8 mm, 8 μm). Detection was performed using ELSD under a nitrogen flow rate of 2 L min^−1^ and a detector temperature of 80 °C [15].

### 2.10. Determination of Alcohol Content by Ebuliometer and pH Determination

The ebuliometer method is based on the fact that individual liquids have different boiling temperatures. Distilled water has a higher boiling point than wine, and the more alcohol a sample contains, the earlier boiling occurs. Before the actual measurement, the ebuliometer had to be verified using a verification solution (78.13 mL of ethanol + distilled water in a 500 mL volumetric flask). After verification, herbal wine samples were determined [17]. The pH of samples was measured by the pH meter Orion 4 STAR.

### 2.11. Sensory Analysis

Samples after 85 days of fermentation were sensorially evaluated by thirteen amateur tasters based on a 100-point wine evaluation system where clarity and color, intensity, purity and harmony of aroma and intensity, purity, harmony and persistence of taste were evaluated. The final results of the sensory analysis have been presented as the global assessment.

### 2.12. Statistics

The measured values were statistically evaluated using the IBM SPSS program via the statistical method one-way ANOVA. Based on the results of Levene’s test, the homogeneity of variances was determined, with the help of which we chose the Tukey test at a value of *p* > 0.05 or the Games–Howell test at a value of Levene’s test of *p* < 0.05.

## 3. Results and Discussion

### 3.1. Antioxidant Capacity Determined by Different Methods

Antioxidant profiles of the infusions and leaves measured by different antioxidant capacity methods are given in Table 3. The highest values of antioxidant capacity were measured in the sample of the raw leaf, which were significantly higher than the values of the infusions. Lower values of the antioxidant activity of the infusions may indicate the insufficient extraction of substances with an antioxidant effect or their thermolabile nature. It is important to note that the use of more suitable extraction methods in further research could have a positive effect on the increase in substances with an antioxidant effect. From the point of view of achieving a higher antioxidant capacity in the final product in further research, it would be necessary to test shorter fermentation times.

A significant difference in antioxidant capacity was also noted when comparing pre-fermented samples with fermented samples of beverage, where the antioxidant capacity values were lower in all samples of the beverage after fermentation, which was confirmed by all the antioxidant capacity methods. The antioxidant profiles of the experimentally produced beverage, measured by different antioxidant capacity methods, are given in Table 4. The first method used to determine the antioxidant capacity was the DPPH method. All samples of herbal beverage showed an almost two-fold decrease in antioxidant capacity compared to samples of infusions before fermentation, shown in Table 3, which could have been caused by the fermentation process. The length of fermentation did not have a huge effect on changes in these values. Differences can be observed in most samples when comparing samples with the same type of yeast (A; AO, B; BO, C; CO), where slightly higher values of antioxidant capacity were measured in all samples with the addition of orange peel and juice. A decrease in antioxidant capacity in a sample of herbal wine compared to the must before fermentation by the DPPH method was also investigated in the study by Panda et al. [18], in which they found overall higher values compared to ours in the sample of herbal wine, as well as in the sample before fermentation. Similar results were also reported in the study by Lan et al. [19], where a decrease in antioxidant capacity was recorded in samples of pomegranate wine, which showed a decreasing trend with an increasing length of fermentation. From the point of view of antioxidant capacity in fermented alcoholic beverages, in future research, it would also be appropriate to observe a longer storage period after the completion of fermentation processes, as such types of products tend to be consumed after a certain length of storage. The effect of storage on the antioxidant capacity of white wine was observed in the study by Kallithraka et al. [20], where they recorded an increase after 3 and 6 months of storage using the DPPH method.

Due to the complexity of food composition, it is difficult to study each antioxidant compound separately. Antioxidant compounds may also undertake synergistic or antagonistic interactions with each other; for instance, gallic acid, the major antioxidant in tea, shows strong antioxidant capacity, but may also act as a pro-oxidant. For that reason, various methods with different mechanisms must be used in parallel to evaluate the antioxidant capacity of beverages, since different methods may give very different results [21]. The results of antioxidant capacity measured by ABTS showed a decrease, with a statistically significant difference (*p* < 0.05) in all samples when comparing samples with 10 and 60 days of fermentation, followed by an increase with a statistically significant difference (*p* < 0.05) in all samples when comparing samples with 60 and 85 days of fermentation. However, this increase did not reach the values measured at the shortest fermentation time. When comparing samples with the same types of yeast, no significant differences were noted.

In almost all samples, the highest value of antioxidant capacity was recorded by the CUPRAC method during fermentation, with a length of 60 days. These values suggest an increase compared to the 10-day fermentation values, with a statistically significant difference (*p* < 0.05) in most samples. Similar to the case of DPPH, a decrease followed in most samples after 60 days of fermentation, but these values in most cases did not fall below the measured values with the shortest fermentation time. However, compared to the pre-fermentation infusions from Table 3, a decrease in the antioxidant capacity was recorded. The effect of fermentation on the antioxidant capacity measured by the CUPRAC method was also investigated in the study by Suna et al. [22], where an increase in the values of antioxidant capacity in kombucha samples was recorded. In this case, however, shorter fermentation times were observed, ranging from 24 to 120 h.

### 3.2. Total Phenolic Content

The highest value of TPC was noted in the sample of fresh leaves before leaching. However, the TPC values of already-leached leaves were significantly lower, which may point to the fact that, in addition to the extraction of polyphenols into the leachate, their degradation may also have occurred due to low thermostability, as the process of pouring boiling water significantly reduced their amount. TPC in oak (*Q. petraeae*) was also investigated by Popović et al. [23], who recorded a three-fold higher result (35.52) in the sample of leaves. However, in this study, TPC was expressed as mg catechin/g. Significantly higher TPC values were recorded by Custódio et al. [24] in samples of methanol and water leaf extracts (*Q. suber*), with values of 211.0 and 61.2 mg GAE/g. Ling et al. [25] stated that the exposure of antioxidants to higher temperatures can cause their degradation. The thermal degradation of polyphenols can occur at different temperatures, but this depends on factors such as pH, extraction time, extraction environment and the source of the material. The extraction temperature also has a significant effect on the types of polyphenols being extracted [26]. For that reason, it would be appropriate for further research to test other extraction methods in order to increase the amount of polyphenols in the final product.

In most samples of fermented beverages, a decrease in the total phenolic content (TPC) was recorded with the increasing length of fermentation, and the lowest TPC values were recorded in all samples (with statistical significance (*p* < 0.05)) at the longest (85 days) period of fermentation, compared to 10-day fermentation (Table 5). A similar decrease in TPC with increasing length of fermentation was also recorded in the study by Székelyhidi et al. [27], who investigated the total phenolic content in a fermented alcoholic beverage made from apples with the addition of herbal extracts. However, this decrease was already noted after a shorter period of fermentation, between the 10th and 17th day of fermentation. Salmon [28] stated that micro-oxygenation caused by yeast can occur during alcoholic fermentation, leading to the formation of reactive oxygen species (ROS), and the presence of these compounds may cause the oxidation of the phenolic compounds present in wine. However, this micro-oxygenation during aging may improve wine quality, and the gentle oxidation of phenolic compounds has a positive effect on the color of the wine and may reduce the astringency of the final product. When comparing the samples of the infusion before and after the boiling process, no significant changes were noted; however, a higher value of TPC was measured in the infusion after boiling with the addition of orange and orange juice.

### 3.3. Phenolic Compounds

The main individual phenolic compounds found in the samples of infusions and leaves are shown in Table 6. In correlation with the values of antioxidant capacity in samples of infusions and leaves, shown in Table 3, the highest values of individual phenolic substances were measured in samples of raw leaves and leaves after leaching compared to the values measured in infusion samples, pointing to the strong antioxidant properties of these substances. For most of the phenolic substances, higher values were recorded in the samples of leached leaf compared to the sample of the raw leaf, which may indicate that the pouring of boiled water and the leaching process had a positive effect on the amount and availability of these substances in the leaf, which, even after leaching, still represented a rich source of phenolic substances. It is interesting to compare the values of some substances in the fresh leaf sample with the infusion samples, whereby we can observe the different extraction abilities of the substances in the infusion. For example, in the case of catechin, the content of which was double that of quercitrin in the raw leaf sample, we can observe lower catechin values in all types of infusions. It is interesting to compare the values of catechin with those measured in the research by García-Villalba et al. [29], where significantly higher values were measured in oak leaf infusions, depending on the species, in the range of 6.61–177 mg/L. The most abundant phenolic compound in leachates before fermentation was chlorogenic acid. Regarding chlorogenic acid, García-Villalba et al. [29] measured significantly lower values in four oak species (*Q. grisea*, *Q. arizonica*, *Q. convallata* and *Q. eduardii*), with values of 0.03–0.38 mg/L. Higher values were observed in *Q. sideroxyla* and *Q. durifolia*, specifically, 21.5 and 33.1 mg/L. It is clear that the number of individual components is strongly influenced by the tree species, and their quantity varies significantly depending on the tree species. The determination of phenolic compounds in oak leaf infusion (*Q. eduardii*, *Q. durifolia*, *Q. sideroxyla* and *Q. resinosa*) was also performed by Rocha-Guzmán et al. [30], where gallic acid, epigallocatechin, vanillin and syringic acid were found to be the most abundant.

The main individual phenolic compounds found in experimentally produced beverages are shown in Table 7 and Table 8. Phenolic compounds are often influenced by several factors, such as temperature, oxygen or processing, which may be related to a decrease in antioxidant capacity due to the degradation of phenols [31]; however, most of the phenolic compounds in the samples showed stability during fermentation, and in some cases, the technological operations of the beverage had a positive effect on their content.

Table 7 shows the most highly represented phenolic substances in the samples of alcoholic beverages, among which chlorogenic acid was the most highly represented in all samples, and its values in the samples were not significantly affected by the different types of yeast or the addition of orange peel and juice. It is interesting to compare the measured values with the values measured by Vázquez-Cabral et al. [8], who reported significantly lower values of chlorogenic acid compared to other phenolic substances such as catechin and gallic acid in samples of fermented beverage from oak leaves. In our samples, catechin was the second most highly represented substance, but its content tended to decrease with increasing fermentation duration in most samples. The other most frequently occurring phenolic compounds recorded in the beverage samples were gallic acid and quercitrin, whose values were relatively stable during the fermentation process, and significant changes in their content were not recorded.

The less highly represented phenolic compounds in the beverage samples were rutin, naringenin, gallocatechin and quercetin. The biggest differences when comparing samples without the addition and with the addition of orange peel and juice were recorded for the content of naringenin; at all lengths of fermentation, a higher content of this phenolic substance was recorded in the samples that contained orange. Naringenin is a substance from the flavonoid group that is widely present in many citrus fruits [32], which explains its higher content in these samples. However, its presence was also recorded in samples without the addition of orange, which points to its natural occurrence in oak leaves. The values of rutin were stable during fermentation, but it is interesting to compare them with the values of rutin in Table 6, where different values were measured depending on the technological treatment.

When comparing the contents of phenolic substances in infusions before fermentation (Table 6) and the contents of phenols in the samples of beverage after fermentation (Table 7 and Table 8), significant increases in the contents of substances such as catechin, gallic acid, gallocatechin and chlorogenic acid can be seen. These results show that the fermentation process had a positive effect on the contents of these substances. However, for some substances, lower values were recorded after the fermentation process, specifically for quercitrin. It is interesting that, for some substances, such as catechin and rutin, very low values were recorded in the infusion, but higher values were recorded after 30 min of boiling. It has been shown that the content of catechin in tea leaves increases after their exposure to sunlight [33]. However, the study by Ross et al. [34] reported that the catechin content in grape seed flour decreased with increasing temperature, but in this study, the catechin was exposed to temperatures higher than 120 °C. These findings show that heat treatment has a positive effect on the catechin content only up to a certain temperature.

In the case of rutin, however, fermentation had the opposite effect on its content, similar to the case of catechin, and the values of rutin after fermentation were lower when measured in the infusion with a 30 min boil but still higher than in the case of infusion. However, the highest concentrations of all phenolic compounds were measured in the fresh leaf sample or in the leaves after leaching, depending on the phenolic substance. For example, in the case of catechin, an increase in its concentration after pouring boiling water was confirmed when comparing leaf samples, where double the concentration was recorded in the leached leaf compared to the raw leaf. Higher concentrations in the leached leaf sample were also recorded for gallocatechin, quercitrin, naringenin and quercetin. The results show the increased bioavailability of these substances in the leaf after heat treatment, but their capacity for extraction into the leachate was still low, and other extraction methods should be chosen in future research to improve it.

### 3.4. Determination of pH

The lowest recorded pH value was found in sample A, with the shortest fermentation time, and the highest pH was recorded in sample CO with the longest fermentation time (Table 9).

The measured values shown in the table are similar to the values of white wine measured in the study by Vahl et al. [35], in which pH was recorded at values of 3.17 and 3.34. Very similar results to ours were recorded in a study by Gamboa-Gómez et al. [36], who observed pH values in the infusion and fermented beverages of *Quercus convallta* leaves (6.02; 3.28) and *Quercus arizonica* (5.42; 3.24). Our results show that 10 days of fermentation was sufficient to cause a significant decrease in pH compared to samples before fermentation, followed by a slight increase in all samples when comparing 10 and 85 days of fermentation. The study by Zeng et al. [37], in which they investigated the effect of pH on the stability of polyphenols extracted from tea, showed that polyphenols were less stable at higher pH, which means that they were more stable in an acidic environment. Therefore, lowering the pH of oak leaf infusions from the original 5.73–5.67 before fermentation to the values obtained after fermentation can have a positive effect on the stability of polyphenolic substances. Opposing results were reported by Forino et al. [38], who claimed that, in the case of anthocyanins, a higher content of these substances occurs in red wine at a lower pH, but in the case of flavon-3-ols, such as catechin, its content is higher at higher pH values. However, this was not confirmed in our case, and the catechin content was higher after fermentation when the pH decreased. It is also good to compare the measured pH values with the values of other herbal wines; for example, in the case of herbal wines made from the biological waste of fruits and vegetables, observed in the study of Deshmukh [12], significantly higher values were recorded—specifically, 5.5 in samples from peels and 5.3 in the samples made from other fruit waste. Due to the oxidation of phenolic substances at higher pH values [39] as well as the effect of pH on the perception of mouthfeel [40], the drop in the pH level in the fermentation process is important.

### 3.5. Sugar Content

After 10 days of fermentation, sucrose was not detected in any sample, and was transformed into glucose and fructose by the yeast. When comparing glucose and fructose contents, higher fructose values were measured in all samples. Berthels et al. [41] reported that the yeast S. cerevisiae prefers glucose during fermentation, leading to higher fructose content in the residual sugar of wines. The biggest changes in the contents of glucose and fructose were recorded between the 10th and 60th days of fermentation, when there was a statistically significant (*p* < 0.05) decrease in all samples due to the ongoing alcoholic fermentation (Table 10). No further decrease in the values of monitored sugars was recorded, from which it is clear that after 60 days, the fermentation processes had ended. According to the results regarding fructose contents in wines reported by Coelho et al. [42], the fructose content in white wine is significantly lower (4.97 g/L), and our measured values correspond more closely to the fructose content of sparkling wine, the values of which are around 35.20–45.12 g/L. Gamboa-Gómez et al. [36] recorded slightly higher values of glucose and fructose in a fermented beverage made from oak leaves, whereas in the case of a sample from *Quercus convallata* leaves, they recorded even higher values of glucose compared to fructose. However, this was a non-alcoholic beverage based on kombucha, where other microorganisms such as Saccharomyces cerevisiae participated in the fermentation process.

### 3.6. Alcohol Content and Sensory Analysis

The alcohol contents in experimentally produced alcoholic beverages, measured using an ebulliometer, are shown in Table 11.

A statistically significant difference (*p* < 0.05) was noted in almost every sample when comparing samples with and without the addition of orange peel and juice. In the samples that contained the addition of orange, a lower sugar content was recorded, regarding both glucose and fructose. These values are related to the higher alcohol contents in these samples, and also with a worse acceptability on the part of assessors due to the less sweet taste of the final product within 85 days of fermentation. The concentration of alcohol in wine has increased in recent years [43], even though a high concentration of ethanol can affect the sensory properties of wine and reduce the complexity of flavors and aromas [44]. Results similar to ours regarding the alcohol content of herbal wines have been reported by Rana and Singh [45], who stated that the alcohol content in herbal wines ranges from 10 to 15%. The higher acceptability on the part of assessors who preferred samples without the addition of the other aromatic components could be associated with the sweetening properties of sugars and their higher contents in these samples. Berthels et al. [41] stated that, due to the high (double) sweetness of fructose compared to glucose, its higher content has a greater influence on the final sweetness of wines. This fact could also have an impact on the final evaluation of the product, since in the beverage samples with a fermentation duration of 85 days (Table 10), double the amount of fructose was recorded in the beverage samples without the addition of orange peel and juice. However, these results also point to the suitability and sufficiency of oak leaves used as a source for the production of fermented alcoholic beverages without the need for additional flavoring components.

## 4. Conclusions

The research conducted underlines the possibilities of producing alcoholic fermented beverages from unused plant raw materials such as oak leaves, which represent a rich source of phenolic substances with an antioxidant effect. For some of these substances, such as catechin, gallocatechin or gallic acid, an increase in concentration was recorded due to the fermentation process compared to the leachates before fermentation. With the increasing length of fermentation, a gradual decrease in the total content of polyphenols was recorded, with statistically significant differences (*p* < 0.05). The beverage samples enriched with the addition of orange peel and orange juice received a lower rating in the sensory analysis, which could be connected to the higher alcohol content and lower residual sugar content in these samples. It could be stated that oak leaves represent a suitable raw material for the production of a fermented beverage, even without the need for the addition of flavoring components. The samples of the beverage contained significantly (*p* < 0.05) lower values of polyphenolic substances as well as antioxidant activity compared to the fresh leaf samples. For that reason, it would be appropriate in future research to investigate other extraction methods, as well as shorter fermentation times, so as to increase the antioxidant properties of the final product.

## Figures and Tables

**Table 1:** Overview of the samples.

Abbreviation	Composition of Herbal Wine Samples
A	yeast BS 2/aromatic
B	yeast BS 2/sekt
C	yeast BS 5/ruland
AO	yeast BS 2/aromatic with orange peel and juice
BO	yeast BS 2/sekt with orange peel and juice
CO	yeast BS 5/ruland with orange peel and juice

**Table 2:** Overview of the samples.

Abbreviation	Composition of Infusions and Leaves Samples
I	Unboiled leaf infusion
BI	Leaf infusion after 30 min of boiling
OBI	Leaf infusion after 30 min of boiling with addition of orange peel and juice
RL	Raw leaves
IL	Infused leaves

**Table 3:** Antioxidant capacities of the infusions and leaves.

Samples	DPPH (%)	ABTS (%)	CUPRAC (Trolox µmol/g)
I	58.59 ± 0.45 ^a^	3.35 ± 0.11 ^a^	6.74 ± 0.01 ^a^
BI	50.01 ± 0.13 ^b^	3.19 ± 0.14 ^a^	5.70 ± 0.02 ^b^
OBI	59.65 ± 0.38 ^a^	3.56 ± 0.11 ^a^	9.02 ± 0.25 ^c^
RL	85.74 ± 0.02 ^c^	40.72 ± 0.24 ^b^	101.44 ± 0.15 ^d^
IL	78.21 ± 0.19 ^d^	9.71 ± 0.16 ^c^	50.30 ± 0.05 ^e^

Explanations: Different lowercase letters in the superscript in the same column indicate a statistically significant difference (*p* < 0.05) between samples.

**Table 4:** Antioxidant capacities of the beverage samples with different lengths of fermentation.

	Samples	A	B	C	AO	BO	CO
DPPH (%)	10 days of fermentation	28.55 ± 0.09 ^aA^	30.98 ± 0.07 ^bA^	31.29 ± 0.97 ^bcA^	31.50 ± 0.10 ^cA^	31.04 ± 0.21 ^bcB^	36.81 ± 0.45 ^dA^
	60 days of fermentation	32.19 ± 0.06 ^afB^	31.92 ± 0.04 ^bB^	31.15 ± 0.07 ^cA^	39.06 ± 0.19 ^dB^	32.51 ± 0.10 ^aeB^	32.01± 0.45 ^bcefB^
	85 days of fermentation	27.52 ± 0.10 ^aC^	29.38 ± 0.04 ^bC^	28.86 ± 0.05 ^cB^	31.06 ± 0.10 ^dC^	32.55 ± 0.08 ^eB^	32.68 ± 0.09 ^eB^
ABTS (%)	10 days of fermentation	2.48 ± 0.08 ^aA^	2.80 ± 0.11 ^aA^	2.94 ± 0.09 ^bA^	2.81 ± 0.04 ^bA^	2.81 ± 0.10 ^bA^	2.82 ± 0.13 ^bA^
	60 days of fermentation	1.90 ± 0.04 ^aB^	1.74 ± 0.04 ^bB^	1.86 ± 0.04 ^abB^	2.12 ± 0.06 ^cB^	2.04 ± 0.02 ^cB^	2.08 ± 0.05 ^cB^
	85 days of fermentation	2.22 ± 0.07 ^aC^	2.21 ± 0.05 ^aC^	2.12 ± 0.07 ^aC^	2.45 ± 0.06 ^bC^	2.49 ± 0.07 ^bC^	2.49 ± 0.09 ^bC^
CUPRAC (Trolox µmol/g)	10 days of fermentation	3.41 ± 0.11 ^abA^	3.74 ± 0.02 ^bA^	3.48 ± 0.02 ^aA^	4.45 ± 0.02 ^cA^	4.02 ± 0.06 ^dA^	3.98 ± 0.01 ^dA^
	60 days of fermentation	4.25 ± 0.08 ^aB^	5.88 ± 0.06 ^bB^	3.82 ± 0.03 ^cB^	5.72 ± 0.06 ^bB^	4.14 ± 0.03 ^aB^	4.00 ± 0.00 ^aA^
	85 days of fermentation	3.31 ± 0.20 ^acA^	4.13 ± 0.11 ^bcC^	4.21 ± 0.02 ^cC^	4.25 ± 0.01 ^cC^	4.10 ± 0.05 ^cAB^	3.86 ± 0.02 ^abB^

Explanations: Different lowercase letters in the superscript on the same line indicate a statistically significant difference (*p* < 0.05) between different samples within the same length of fermentation. Different uppercase letters in the superscript in the same column indicate a statistically significant difference (*p* < 0.05) within the same sample under the different length of fermentation separately for each determination.

**Table 5:** Total phenolic contents of the beverage, infusions and leaves.

Total Phenolic Content (mg GAE/g)					
Samples	10 Days of Fermentation	60 Days of Fermentation	85 Days of Fermentation	Samples	
A	0.26 ± 0.00 ^aA^	0.24 ± 0.00 ^aB^	0.20 ± 0.01 ^aC^	I	0.26 ± 0.01 ^a^
B	0.30 ± 0.00 ^bA^	0.31 ± 0.03 ^abdeA^	0.20 ± 0.01 ^aB^	BI	0.25 ± 0.00 ^a^
C	0.28 ± 0.00 ^cdA^	0.25 ± 0.00 ^bB^	0.23 ± 0.01 ^abB^	OBI	0.34 ± 0.00 ^b^
AO	0.29 ± 0.00 ^cdA^	0.37 ± 0.00 ^cdB^	0.22 ± 0.00 ^bcC^	RL	11.87 ± 0.00 ^c^
BO	0.28 ± 0.00 ^dA^	0.28 ± 0.01 ^beA^	0.22 ± 0.00 ^acB^	IL	2.75 ± 0.00 ^d^
CO	0.29 ± 0.00 ^bcA^	0.29 ± 0.00 ^eA^	0.21 ± 0.00 ^acB^	-	-

Explanations: Different lowercase letters in the superscript in the same column indicate a statistically significant difference (*p* < 0.05) between different samples with the same length of fermentation. Different uppercase letters in the superscript on the same line indicate a statistically significant difference (*p* < 0.05) within the same sample and different lengths of fermentation.

**Table 6:** Values of the individual phenolic compounds in infusions and leaves.

Concentration	mg/L			mg/kg	
Samples	I	BI	OBI	RL	IL
catechin	0.00 ± 0.00 ^a^	1.40 ± 0.21 ^a^	0.77 ± 0.06 ^a^	99.82 ± 13.56 ^b^	200.03 ± 17.58 ^c^
gallic acid	0.016 ± 0.00 ^a^	0.14 ± 0.02 ^a^	0.06 ± 0.01 ^a^	32.66 ± 2.91 ^b^	7.45 ± 1.80 ^a^
gallocatechin	ND	0.3 ± 0.00 ^a^	ND	2.06 ± 0.22 ^b^	9.72 ± 0.88 ^c^
chlorogenic acid	0.18 ± 0.00 ^a^	6.14 ± 0.25 ^b^	2.70 ± 0.14 ^c^	ND	ND
quercitrin	1.50 ± 0.01 ^a^	1.53 ± 0.24 ^a^	1.54 ± 0.07 ^a^	46.11 ± 5.14 ^b^	96.71 ± 1.27 ^c^
naringenin	0.02 ± 0.00 ^a^	0.58 ± 0.10 ^abc^	0.23 ± 0.00 ^b^	3.25± 0.32 ^c^	6.12 ± 0.72 ^d^
rutin	0.04 ± 0.00 ^a^	1.15 ± 0.16 ^ab^	0.87 ± 0.01 ^b^	21.70 ± 2.38 ^c^	18.11 ± 0.84 ^c^
quercetin	ND	0.02 ± 0.00 ^a^	ND	0.47 ± 0.06 ^b^	1.49 ± 0.10 ^c^

Explanations: Different lowercase letters in the superscript on the same line indicate a statistically significant difference (*p* < 0.05) between samples within one specific compound. ND—not detected.

**Table 7:** Values of the individual phenolic compounds in the beverage samples.

Concentration (mg/L)					
Samples		Catechin	Gallic Acid	Quercitrin	Chlorogenic Acid
A	10 days of fermentation	1.32 ± 0.13 ^aA^	0.30 ± 0.02 ^aA^	0.96 ± 0.05 ^aA^	2.58 ± 0.15 ^aAB^
B		1.66 ± 0.10 ^aA^	0.44 ± 0.02 ^bA^	0.87 ± 0.07 ^aA^	2.95 ± 0.13 ^aA^
C		1.90 ± 0.15 ^aA^	0.48 ± 0.03 ^bA^	0.92 ± 0.01 ^aA^	2.90 ± 0.19 ^aA^
AO		1.77 ± 0.10 ^aA^	0.38 ± 0.03 ^baA^	0.96 ± 0.00 ^aA^	2.84 ± 0.08 ^aA^
BO		1.83 ± 0.05 ^aA^	0.45 ± 0.02 ^bA^	0.85 ± 0.05 ^aA^	2.67 ± 0.16 ^aA^
CO		1.57 ± 0.09 ^aA^	0.44 ± 0.02 ^bA^	1.02 ± 0.05 ^aA^	2.33 ± 0.07 ^aA^
A	60 days of fermentation	1.30 ± 0.03 ^abA^	0.32 ± 0.03 ^acA^	0.94 ± 0.06 ^abA^	2.55 ± 0.00 ^aA^
B		1.51 ± 0.03 ^abA^	0.53 ± 0.01 ^bA^	0.93 ± 0.05 ^abA^	3.01 ± 0.07 ^aA^
C		1.40 ± 0.01 ^bAB^	0.51 ± 0.08 ^bA^	0.83 ± 0.00 ^abA^	2.27 ± 0.24 ^aA^
AO		1.54 ± 0.01 ^aA^	0.22 ± 0.02 ^aB^	0.92 ± 0.03 ^aA^	2.44 ± 0.01 ^aAB^
BO		1.48 ± 0.56 ^abA^	0.44 ± 0.01 ^cbA^	0.92 ± 0.06 ^abA^	2.55 ± 0.03 ^aA^
CO		1.55 ± 0.01 ^aA^	0.36 ± 0.01 ^acA^	1.18 ± 0.02 ^bA^	2.46 ± 0.08 ^aA^
A	85 days of fermentation	1.04 ± 0.01 ^aA^	0.32 ± 0.00 ^acA^	0.93 ± 0.01 ^aA^	2.69 ± 0.01 ^aB^
B		1.20 ± 0.12 ^abcA^	0.55 ± 0.06 ^bA^	0.95 ± 0.18 ^aA^	3.18 ± 0.15 ^aA^
C		0.96 ± 0.09 ^abcB^	0.47 ± 0.03 ^abA^	1.00 ± 0.02 ^aA^	2.25 ± 0.17 ^aA^
AO		1.32 ± 0.02 ^bA^	0.19 ± 0.05 ^cAB^	0.92 ± 0.05 ^aA^	2.21 ± 0.09 ^aB^
BO		1.80 ± 0.03 ^cA^	0.48 ± 0.00 ^abA^	0.97 ± 0.02 ^aA^	2.69 ± 0.09 ^aA^
CO		1.34 ± 0.18 ^abcA^	0.37 ± 0.25 ^adA^	1.07 ± 0.09 ^aA^	2.63 ± 0.10 ^aA^

Explanations: Different lowercase letters in the superscript in the same column indicate a statistically significant difference (*p* < 0.05) between samples for each fermentation length. Different uppercase letters in the superscript in the same column indicate a statistically significant difference (*p* < 0.05) between the fermentation lengths within the same sample.

**Table 8:** Values of the individual phenolic compounds in the beverage samples.

Concentration (mg/L)					
Samples		Gallocatechin	Naringenin	Rutin	Quercetin
A	10 days of fermentation	0.16 ± 0.01 ^aAB^	0.16 ± 0.02 ^aA^	0.59 ± 0.04 ^aA^	0.01 ± 0.00 ^aA^
B		0.25 ± 0.01 ^bA^	0.20 ± 0.01 ^aA^	0.59 ± 0.02 ^aA^	0.01 ± 0.00 ^aA^
C		0.20 ± 0.00 ^abA^	0.37 ± 0.01 ^bA^	0.64 ± 0.04 ^aA^	0.01 ± 0.00 ^aA^
AO		0.20 ± 0.01 ^abA^	0.36 ± 0.06 ^abA^	0.67 ± 0.04 ^aA^	0.00 ± 0.00 ^aA^
BO		0.23 ± 0.01 ^abAB^	0.33 ± 0.02 ^abA^	0.61 ± 0.02 ^aA^	0.01 ± 0.00 ^aA^
CO		0.20 ± 0.00 ^abA^	0.43 ± 0.04 ^abA^	0.61 ± 0.01 ^aA^	0.01 ± 0.00 ^aA^
A	60 days of fermentation	0.12 ± 0.01 ^adB^	0.17 ± 0.03 ^aA^	0.54 ± 0.02 ^aA^	0.01 ± 0.00 ^aAB^
B		0.17 ± 0.00 ^bA^	0.20 ± 0.00 ^aA^	0.62 ± 0.00 ^aA^	0.01 ± 0.00 ^bB^
C		0.12 ± 0.01 ^acAB^	0.27 ± 0.03 ^abA^	0.53 ± 0.04 ^aA^	0.01 ± 0.00 ^abA^
AO		0.10 ± 0.01 ^cB^	0.26 ± 0.03 ^abA^	0.62 ± 0.01 ^aA^	0.01 ± 0.00 ^aB^
BO		0.14 ± 0.00 ^aA^	0.40 ± 0.14 ^abA^	0.60 ± 0.59 ^aA^	0.00 ± 0.00 ^aA^
CO		011 ± 0.00 ^cdB^	0.49 ± 0.01 ^bA^	0.63 ± 0.00 ^aA^	0.01 ± 0.00 ^abB^
A	85 days of fermentation	0.17 ± 0.00 ^aA^	0.20 ± 0.02 ^aA^	0.59 ± 0.00 ^aA^	0.01 ± 0.00 ^aB^
B		0.19 ± 0.01 ^aA^	0.21 ± 0.01 ^aA^	0.60 ± 0.07 ^abA^	0.01 ± 0.00 ^abcdB^
C		0.12 ± 0.00 ^bB^	0.33 ± 0.08 ^abA^	0.50 ± 0.01 ^aA^	0.01 ± 0.00 ^bA^
AO		0.07 ± 0.00 ^cC^	0.33 ± 0.06 ^abA^	0.64 ± 0.05 ^abA^	0.00 ± 0.00 ^cA^
BO		0.12 ± 0.00 ^bB^	0.37 ± 0.02 ^bA^	0.67 ± 0.01 ^bA^	0.01 ± 0.00 ^dB^
CO		0.09 ± 0.01 ^cB^	0.51 ± 0.05 ^abA^	0.65 ± 0.06 ^abA^	0.01 ± 0.00 ^abcdAB^

Explanations: Different lowercase letters in the superscript in the same column indicate a statistically significant difference (*p* < 0.05) between samples for each length of fermentation. Different uppercase letters in the superscript in the same column indicate a statistically significant difference (*p* < 0.05) between the fermentation lengths within the same sample.

**Table 9:** pH values of the beverages and infusions.

Samples	10 Days of Fermentation	60 Days of Fermentation	85 Days of Fermentation	Samples	pH
A	3.20 ± 0.01 ^adA^	3.22 ± 0.01 ^aA^	3.29 ± 0.01 ^aA^	I	5.73 ± 0.01 ^a^
B	3.41 ± 0.01 ^bcefA^	3.38 ± 0.00 ^cdA^	3.44 ± 0.00 ^abcA^	BI	5.67 ± 0.01 ^b^
C	3.34 ± 0.00 ^cefgA^	3.51 ± 0.00 ^bdAB^	3.5 ± 0.01 ^bB^	OBI	5.64 ± 0.02 ^b^
AO	3.28 ± 0.01 ^dghA^	3.37 ± 0.01 ^cA^	3.53 ± 0.01 ^bB^	-	-
BO	3.52 ± 0.02 ^efA^	3.56 ± 0.01 ^bdA^	3.62 ± 0.00 ^bcA^	-	-
CO	3.39 ± 0.01 ^fhA^	3.59 ± 0.01 ^bB^	3.67 ± 0.00 ^cB^	-	-

Explanations: Different lowercase letters in the superscript in the same column indicate a statistically significant difference (*p* < 0.05) between samples. Different uppercase letters in the superscript on the same line indicate a statistically significant difference (*p* < 0.05) between samples.

**Table 10:** Sugar contents in the beverage samples.

	Glucose (g/L)		Fructose (g/L)			
Samples	10 Days of Fermentation	60 Days of Fermentation	85 Days of Fermentation	10 Days of Fermentation	60 Days of Fermentation	85 Days of Fermentation
A	41.32 ± 0.10 ^aA^	12.59 ± 0.37 ^abB^	11.71 ± 0.06 ^aB^	91.65 ± 0.11 ^aA^	48.01 ± 0.73 ^aB^	39.70 ± 1.40 ^acdB^
B	31.07 ± 0.07 ^bA^	12.13 ± 0.19 ^aB^	11.26 ± 0.03 ^aB^	70.35 ± 0.33 ^beA^	30.12 ± 1.67 ^abcdeB^	29.70 ± 0.28 ^aB^
C	39.34 ± 0.11 ^cA^	7.8 ± 0.06 ^bB^	7.86 ± 0.03 ^bB^	87.73 ± 0.14 ^ceA^	31.25 ± 0.07 ^cdB^	32.22 ± 1.47 ^abB^
AO	18.79 ± 0.57 ^dA^	3.3 ± 0.04 ^cB^	3.2 ± 0.02 ^cB^	57.79 ± 0.33 ^deA^	19.48 ± 1.00 ^bdeB^	20.00 ± 0.67 ^bB^
BO	19.51 ± 1.23 ^bcdA^	2.80 ± 0.00 ^cdB^	2.81 ± 0.11 ^cdB^	48.62 ± 3.12 ^eA^	15.55 ± 0.04 ^bB^	15.51 ± 0.01 ^bdB^
CO	18.19 ± 0.09 ^dA^	2.02 ± 0.05 ^dB^	2.03 ± 0.03 ^dB^	49.20 ± 0.20 ^feA^	17.33 ± 0.20 ^eB^	17.30 ± 0.16 ^bcB^

Explanations: Different lowercase letters in the superscript in the same column indicate a statistically significant difference (*p* < 0.05) between samples. Different uppercase letters in the superscript on the same line indicate a statistically significant difference (*p* < 0.05) between samples for each sugar.

**Table 11:** Alcohol content and sensory analysis of beverage.

	Alcohol Content (% vol.)	Sensory Analysis (Global Assesment)
Samples	85 Days of Fermentation	85 Days of Fermentation
A	11.75 ± 0.14 ^a^	84.54 ± 8.82 ^a^
B	12.10 ± 0.27 ^ab^	77.00 ± 7.51 ^ab^
C	12.38 ± 0.10 ^ab^	75.54 ± 10.97 ^ab^
AO	12.18 ± 0.14 ^ab^	77.92 ± 10.68 ^ab^
BO	12.74 ± 0.04 ^b^	72.62 ± 9.00 ^b^
CO	12.90 ± 0.40 ^b^	71.08 ± 5.88 ^b^

Explanations: Different lowercase letters in the superscript in the same column indicate a statistically significant difference (*p* < 0.05) between samples.

## Data Availability

The original contributions presented in the study are included in the article, further inquiries can be directed to the corresponding author.
